# 40977 Assessing the Need for Competency-Based Self-Assessment Tools for CTSA Professionals

**DOI:** 10.1017/cts.2021.555

**Published:** 2021-03-30

**Authors:** Peter Trinh, Barbara Tafuto, Yasheca Ebanks, Zahra Zunaed, Doreen W. Lechner

**Affiliations:** 1 Rutgers Robert Wood Johnson Medical School, Rutgers, The State University of New Jersey; 2 New Jersey Alliance for Clinical and Translational Science, Department of Health Informatics, School of Health Professions, Rutgers, The State University of New Jersey; 3 Ernest Mario School of Pharmacy, Rutgers, The State University of New Jersey

## Abstract

ABSTRACT IMPACT: This study works to improve the quality of clinical and translational workforce development programs in order to enhance the training of researchers in the field. OBJECTIVES/GOALS: Evaluating the impact of Clinical and Translational Science Awards (CTSA) Programs is crucial. To this end, the value of competency-based metrics to assess the professional growth of CTSA awardees is unknown. A needs assessment was conducted to determine the present use and potential need for a competency-based self-assessment tool. METHODS/STUDY POPULATION: A mixed methods study was conducted using synchronous live interviews and asynchronous online surveys. Study authors contacted 102 CTSA administrators nationwide for live interviews according to I-Corps ******„¢Customer Discovery Guidelines. Interviews were recorded and transcribed through Innovation Within, an I-Corps „¢online platform and independently analyzed by two members of the study team. An online REDCap survey was also distributed to 63 CTSA hubs via an internal listserv. In an attempt to elicit responses similar to the I-Corps „¢Customer Discovery Guidelines, the survey asked questions related to the use of competency assessments and requested explanatory responses but did not explicitly ask respondents if they needed a competency-based self-assessment tool. RESULTS/ANTICIPATED RESULTS: Overall, 30 unique CTSA hubs participated. Interview requests and surveys had a response rate of 22% (22 out of 102) and 33% (21 out of 63), respectively. Of the interviewees, 32% (7 out of 22) reported existing use of a competency-based assessment tool, and 59% (13 out of 22), inclusive of those already using a tool, indicated a clear need for one. Of the survey respondents, 62% (13 out of 21) already use a CBST. Interviewees highlighted preferred features for a CBST: customization, soft skills assessment, and integration with local academic institutions. Communication and teamwork were highly valued soft skills, a finding reinforced by survey results in which 80% of respondents marked oral and written communication and teamwork as important skills for their professional workforce. DISCUSSION/SIGNIFICANCE OF FINDINGS: Among CTSA administrators involved with workforce development, there is notable interest in a competency-based self-assessment tool, particularly one that is customizable, soft skill-focused, and integrated with local educational systems.

